# Modelling the Distribution and Habitat Suitability of the European Wildcat (*Felis silvestris*) in North-Western Spain and Its Conservation Implications

**DOI:** 10.3390/ani14182708

**Published:** 2024-09-18

**Authors:** Pablo Vázquez García, Alejandra Zarzo-Arias, Efrén Vigón Álvarez, Iván Alambiaga, Juan S. Monrós

**Affiliations:** 1 Cavanilles Institute of Biodiversity and Evolutionary Biology (ICBIBE), University of Valencia, 46980 Paterna, Spain; pavagar@gmail.com (P.V.G.); ivan.alambiaga@uv.es (I.A.); 2 Department of Biology, Universidad Autónoma de Madrid, c/Darwin 2, 28049 Madrid, Spain; alejandra.zarzo@gmail.com; 3 Department of Organisms and Systems Biology, Universidad de Oviedo, c/San Francisco, 33071 Oviedo, Spain; 4 Laboratorio de Sanidad Vegetal, Gobierno del Principado de Asturias, C/Lucas Rodríguez Pire Nº 4, 33011 Oviedo, Spain; efvigon@gmail.com

**Keywords:** habitat availability, human conflicts, MaxEnt, carnivore

## Abstract

**Simple Summary:**

Human activities have led to significant global habitat degradation and fragmentation. However, some carnivores have adapted to these conditions and are expanding, leading to closer coexistence with humans and potential conflicts. This study analysed over 350 sightings of the European wildcat (*Felis silvestris*) in NW Spain over 17 years to develop suitability models based on environmental, topographic, climatic, and human impact factors. Using MaxEnt, the study predicted the species’ potential regional distribution. The results revealed that less than a third of the suitable areas for wildcats had confirmed their presence. Elevation, forested area percentage, and footpath density were key factors influencing wildcat presence, with the first two having positive effects and footpath density having a negative impact. The wildcats’ preference for high and forested areas likely relates to food availability, while avoiding footpaths is linked to human-related mortality. These findings provide insights for conservation strategies to protect the species.

**Abstract:**

Human activities have resulted in severe habitat degradation and fragmentation at a global scale. Despite this scenario, some carnivore species that adapted to the new conditions are expanding, leading to close coexistence with humans and the emergence of potential conflicts. In this work, we used a European wildcat (*Felis silvestris*) observations database of more than 350 sightings over 17 years in NW Spain to build suitability models based on environmental, topographic, climatic, and human impact variables. MaxEnt was used to analyse the availability of suitable habitats for the species at a regional scale. Our results showed that less than one third of the suitable area for the species had confirmed wildcat presence. Elevation, the percentage of forested area, and footpath density were the three main variables conditioning wildcat presence, with the first two variables having positive effects and footpath density negatively affecting wildcat presence. The selection of high areas and forest areas by the species seems to be related to food availability, while the avoidance of footpaths seems to be related to the fact that main mortality causes are linked to human disturbances. The results enhance the understanding of the European wildcat ecology and provide insight into potential management plans to ensure the conservation of one of the main populations of the species throughout its range.

## 1. Introduction

In the global context of widespread habitat fragmentation, many large carnivores have adapted to human-modified landscapes and have well-documented population expansions, which can lead to various types of conflicts with humans [1,2]. Potential mesocarnivore population expansions, however, remain poorly known, especially in nocturnal, small–medium sized species that are difficult to monitor [3]. Predicting potential areas of range expansion is an important step towards minimising potential conflicts and implementing effective management strategies [4]. Habitat suitability models are commonly used to explore the availability of favourable habitats and the spatial occurrence of potential carnivore population expansion areas [5,6,7]. Different habitat suitability approaches, like maximum entropy models [8], have been used for analysing the availability of favourable habitats in a region, the probable direction of population expansions, and the environmental factors determining them [9,10]. Since reliable absence data are frequently difficult to obtain in this group, maximum entropy models have proven to provide accurate predictions based only on species presence data [11].

The European wildcat (*Felis silvestris*) was once widely distributed across Europe, only absent in Scandinavia, but between 1700 and the mid-1900s, there was a severe decline of populations mainly driven by habitat fragmentation and human persecution [12,13,14]. This resulted in a severe geographical contraction of the species, which led to the patchy distribution across Europe that can be found today. Population decline is still ongoing in some areas of its range, where low food availability, hybridisation with domestic cats, habitat fragmentation, and human activities remain a threat to the European wildcat [14,15]. In other areas of Europe, such as Italy, Germany, France, or Austria, however, population increases have been recorded [16,17]. A decrease in human persecution after the species was listed as protected by the Bern Convention and the EU Habitats Directive, and the low hybridisation rate with the domestic cat in areas with stable wildcat densities [15,18] seem to be some of the reasons for this recovery. In addition, recent studies on habitat requirements suggest that in some parts of its range, this species benefits from mosaic systems including natural forests and agricultural patches, where its main prey are abundant and where it is frequently observed hunting [19,20].

Many studies focusing on the different ecological parameters of European wildcats have been conducted in recent years, including diel activity patterns [21,22], habitat requirements [23], interactions with other sympatric species [24], spatial ecology [19], behaviour [25], or diet [26]. Nevertheless, potential expansion areas facing possible population increases and their implications have not been previously assessed. Spain holds one of the main populations of the European wildcat, with different population trends registered across the country [27,28]. North west Spain sustains one of the most diverse mammal–carnivore communities in Europe with some species clearly expanding and conflicting with human presence [9,29]; however, the status of European wildcat populations is poorly known in the region.

This study is particularly compelling due to the limited knowledge about the status of the wildcats in the northern Iberian Peninsula and their habitat requirements. Identifying potential areas for the species’ expansion is crucial for the development of effective conservation plans. In this research, we employ maximum entropy models to pinpoint suitable habitats for wildcats. Specifically, we (1) identified habitats at a 3 × 3 km scale and (2) evaluated which environmental variables determine habitat suitability for wildcats in Asturias.

## 2. Methods

### 2.1. Study Area

**Figure 1:**
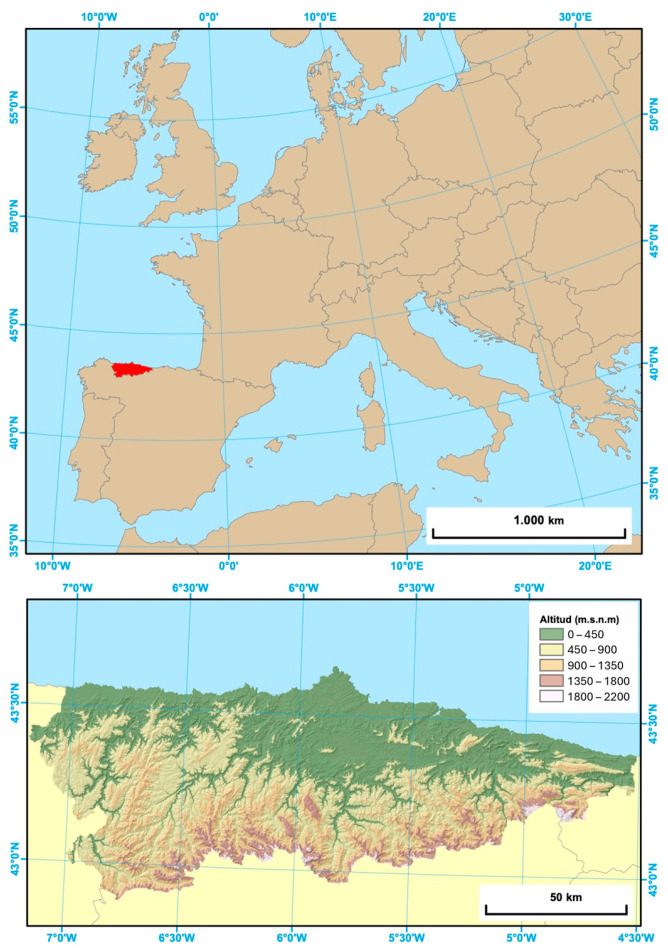
Location of the study area, Asturias, Spain.

The study area was the Principality of Asturias, a region located in northwestern Spain (Figure 1). Asturias has an area of 10,602 km^2^, with more than one million inhabitants (population density ca. 100 inhabitants/km^2^; Instituto Nacional de Estadística, http://www.ine.es/jaxiT3/Datos.htm?t=2886 (accessed on 1 September 2024)) and a road density of 47.4 km/100 km^2^ (http://www.seap.minhap.gob.es/index.html (accessed on 1 September 2024)). The region is characterised by an oceanic climate with mean temperatures ranging from 14 °C on the coast to 2–3 °C in the highest regions of the Cantabrian Mountains (http://www.worldclim.org/). Maximum elevation is 2648 m.a.s.l. in Picos de Europa National Park. Asturias has approximately 46% forest cover, mainly chestnut (*Castanea sativa*), oak (*Quercus petraea*, *Q. pyrenaica*, and *Q. rotundifolia*), and beech (*Fagus sylvatica*), interspersed with pastures and scrublands, and subalpine scrubland above 1700 m [30].

### 2.2. Wildcat Occurrence Data, Definition of Wildcat Range, and Characterisation of Suitable Habitats Available

**Figure 2:**
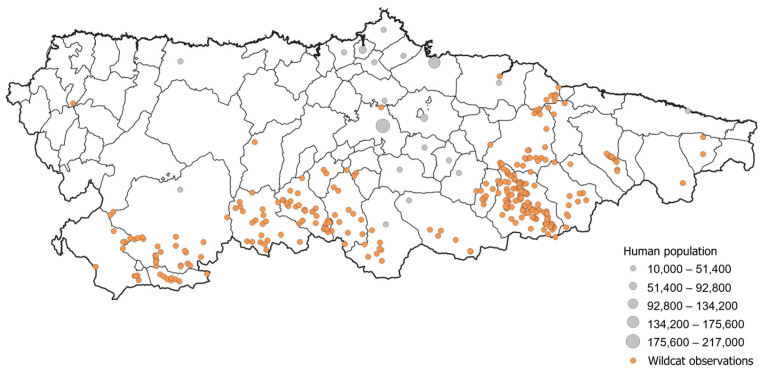
Wildcat observations during the study period (2000–2017) in Asturias and municipalities with more than 10,000 inhabitants in the region. Dot sizes reflect the population size. Information about municipalities’ inhabitants extracted from the National Statistics Institute (INE).

For this study, a database of 350 georeferenced wildcat observations collected from 2000 to 2017 and compiled by the regional government of the Principality of Asturias was used (Figure 2). Data came from direct observations of live animals throughout the day by both the government field staff and the authors. Staff collecting data were experts in carnivore identification, but to prevent potential misidentification with domestic cats and hybrids, only sightings where two observers were absolutely confident that the sighting was a wildcat were considered. In addition, hybridisation between wildcats and domestic cats in areas with high densities of the former, such as the study area, is very low [15], and distinguishing between wildcats and domestic cats is entirely possible for trained staff when conditions in the field are optimal.

Because the overall density of European wildcats in Asturias is unknown, and because observations were more abundant in protected areas where field staff is more frequent, potential sampling biases were corrected by subsampling the dataset. To do this, a 3 × 3 km grid (9 km^2^) was super-imposed over the study area and only one record per grid cell was selected [31], resulting in 137 wildcat presence points. Habitat availability was then evaluated by using the same grid size, corresponding to the approximate size of European wildcat home ranges in Spanish populations [32]. We opted to model the suitability for distribution range rather than for occurrence [33] to obtain a general distribution of the favourable habitat for this species. The aim was to classify each grid either as suitable or unsuitable area.

### 2.3. Environmental Variables

A total of 29 variables related to human infrastructure, vegetation, climate, and topography were used as predictors in habitat suitability models (Table 1). Variable groups were chosen based on previous studies in the Iberian Peninsula and the rest of Europe, testing the importance of habitat type [20,34], topography [35], human disturbances [25], and climate and productivity metrics [36] on the abundance of this species. Climate variables including annual mean temperature, max temperature of the warmest month, min temperature of the coldest month, temperature annual range, annual precipitation, and precipitation seasonality were extracted from the WorldClim database (www.worldclim.org). Discrete land cover types came from Cartografía Temática Ambiental del Principado de Asturias 1989–1998 (1:50,000), which was converted into percent area of each habitat type within each pixel of the 3 × 3 km grid. Any land cover class that did not possess at least a 1% mean occurrence was discarded. Each vegetation class was separated into different layers in our models, with each layer describing the relative abundance of a particular class. The number of land cover classes per pixel and Shannon index of relative abundances were then calculated. Normalised Difference Vegetation Index (NDVI; a measure of greenness), provided by the Instituto de Recursos Naturales y Ordenacion del Territorio (INDUROT), was used as an index of primary productivity. Elevation and mean aspect of the slopes was extracted from a digital elevation model (MDT200) by the Instituto Geográfico Nacional (http://www.sadei.es/es/portal.do (accessed on 14 November 2018)). Finally human population, highway, road, footpath, and river density were obtained from geophysical layers maintained by the Principality of Asturias and from Sociedad Asturiana de Estudios Económicos e Industriales (http://www.sadei.es/es/portal.do (accessed on 14 November 2018)).

When any two variables showed a high spatial correlation > 0.7 (Pearson coefficient), the variable that was considered most relevant for wildcat biology for the authors was kept. High correlation was only found between most climate variables and elevation. All climatic variables except seasonal variability in precipitation were removed, since elevation was considered more ecologically relevant for wildcats as found in previous works [35,36,37]. A total of 24 uncorrelated variables were used for further analysis (Table 1). All variables were projected to the same reference system (ETRS89/ETRS-TM29) and scaled to a 3 × 3 km resolution. Cells that had less than 50% of their area within Asturias were removed from the final model.

### 2.4. MaxEnt Modelling

To model availability of suitable habitats in the study area, MaxEnt v.3.4.4 [38] was executed in R v.3.3.3 using the “dismo” v. 1.0-12 [39] and “ENMeval” v.0.2.0 [40] packages. MaxEnt calculates the probability distribution of species occurrence on the basis of the constraints derived from environmental data at locations where the study species was present. We had 339 wildcat observation locations clustered into 137 pixels of a known wildcat range that were used as training locations for the model. The geographic coordinate assigned to each cell was chosen to be the midpoint of each cell. In total, 500 iterations, using a convergence threshold of 10-5, were performed with values from all cells in the study region as background points to build the model. Model output was projected onto the entire region of Asturias once an optimal model structure had been selected.

### 2.5. Selecting and Evaluating Models and Variable Contributions

MaxEnt associates the presence data with environmental values using five feature types (linear, quadratic, product, threshold, and hinge), representing different types of parameterisations. Software controls overfitting was performed using a regularisation parameter, penalising variables with low contribution to the model. Although machine learning algorithms such as the one used in MaxEnt generally favour more complex model solutions than likelihood-based models, overfitting can still be problematic since models essentially will parameterise random spatial noise [41]. To identify an optimal model structure, we evaluated candidate models with all types of feature combinations, each run over a set of regularisation multipliers ranging from 0 to 10 (Supporting Information Figure S1). Each model included the same set of 24 uncorrelated environmental variables. The best combination of features and regularisation multipliers were identified using the Akaike’s Information Criterion corrected for small sample sizes (AICc) [42]. AICc values were calculated from the raw model output where the sum of the log transformed raw values were treated as the equivalent of model likelihood [41]. AICc values have been shown to be an efficient and reliable method for identifying optimal levels of model complexity in MaxEnt applications. Following [43], we regarded models within 2 AICc units of each other as having equivalent empirical support. Of these models, the model containing the least number of parameters for each spatial scale was selected, and if two models had the same number of parameters, the one with the lowest number of feature types was chosen.

Model performance was evaluated in two ways. First, an area under a receiver operating characteristic curve (AUC) value [44], indicating how efficiently a model differentiates between occurrence and background locations, was used. AUC values from 0.7 to 1 generally suggest that the model has adequate predictive ability [45]. Second, three metrics based on cross-validations were used. A checkerboard method was firstly used to separate occurrence data into one training and one testing bin [40]. Based on this separation, the three metrics were calculated as further indicators of model performance: (1) AUCtest, a threshold-independent metric that describes the ability of testing locations to distinguish between background and presence locations; (2) AUCdiff, a threshold-independent metric that describes the difference in the ability to distinguish between presence and background locations between training and test data [41]; and (3) ORmin (Minimum Omision Rate), a threshold-dependent metric that shows the proportion of test locations with a value below the lowest value of training locations [40].

To evaluate the relative contribution of each environmental variable to the model, jackknife and heuristic methods provided by MaxEnt were used [46] (Supporting Information Figure S2). The jackknife test shows the gain in AUC value of each variable when used in isolation and the lack of gain when removed from the full set of variables (Supporting Information Figure S2). The heuristic method calculates the percent contribution of each variable as the proportional contribution to the model training gain for every iteration of the model fitting process [46].

### 2.6. Model Output

**Figure 3:**
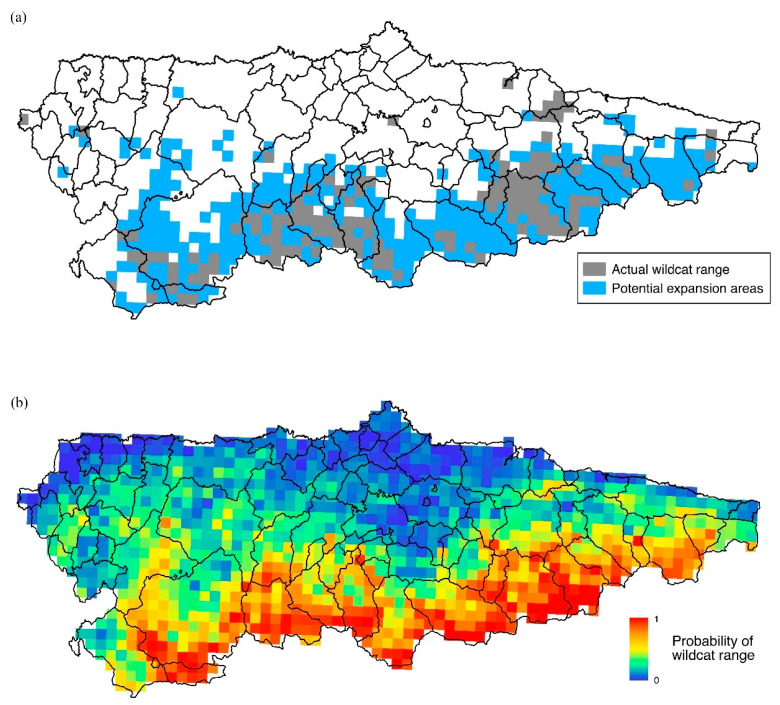
(**a**) Actual and potential wildcat distribution areas based on our Maxent model and (**b**) probability of wildcat range occurrence in Asturias based on our Maxent model using 3 × 3 km cell.

Complementary log-log (cloglog) format was used as model output, as it has an intuitive interpretation and is monotonically related to other potential output formats [8]. This format allows to interpret model output as a probability of occurrence. However, as we were interested in suitable areas for wildcat range in our model, and not necessarily the relative suitability within an identified range, the model was presented as a binary classification, which broadly identifies favourable wildcat range. For this purpose, we used the 10th percentile training presence in the cloglog values as a threshold for suitable European wildcat range. This threshold selects the value above which 90% of the training locations are correctly classified and is one of the most common thresholds used in MaxEnt habitat suitability models [47]. However, the unclassed cloglog output model is also presented in Figure 3.

## 3. Results

### 3.1. Maxent Model Selection and Evaluation

The best-fit model included linear, quadratic, hinge, and threshold features and a regularisation multiplier of 1.5. The model had a mean AUC value of 0.79 (Table 2). More complex models evaluated here generally showed signs of overfitting, whereas less complex models lost predictive abilities (Supporting information Figure S1). The 10th percentile method yielded a threshold of 0.43 in cloglog values, which we used to define suitable areas for wildcat range.

### 3.2. Predicted Range Distribution and Potential Expansion Areas

Wildcats are currently present on 1233 km^2^ of the total Asturias surface. Most wildcat range occurs in the southern part of the province and our model accurately reflects this distribution. The best model selected identified 3953 km^2^ as suitable wildcat range, which represents about 40% of Asturias’ territory. The results showed that only 30% of the potential European wildcat territories in the region are currently occupied (Figure 3).

### 3.3. Environmental Variable Contributions to the Model

The three variables that contributed the most to our model were elevation, the percentage of forested area, and footpath density (Table 3 and Supporting information Figure S2). Elevation and the percentage of forested area showed a clear positive effect on the suitability for European wildcat distribution while footpath density had a negative effect (Supporting information Figure S3) but contributed modestly to the model. The variable contribution was confirmed by jackknife and heuristic evaluations (Table 3 and Supporting information Figure S2).

## 4. Discussion

The modelling results suggest that a large part of the study area is suitable for the European wildcat but less than one third is occupied, providing valuable insights into potential expansion opportunities in the case of population growth. Our findings on the factors influencing the species distribution agree with previous wildcat studies, where models indicated positive correlations with elevation and forested areas, and negative correlations with proximity to roads and human population density [25,35,48]. No sightings were recorded in the most populated areas, as shown in Figure 2. Direct persecution by humans because they were considered a competitor for hunting activities, damaging small-game species, is one of the main causes of threat to the European wildcat in the Iberian Peninsula [49]. For the same reason, the expanded use of poisoned baits from the 1990s and onwards was a major cause of the mortality of the species [50]. In addition, the increased frequency of big game hunting seems to have a strong negative effect on wildcats in the north of Spain [51]. Given that hunters often use the footpaths quantified in our model, hunting may be one potential reason causing the negative association between footpaths and wildcat presence in our models. Human activities not related to hunting can also have a significant effect on European wildcat behaviour, involving more time on alert and less time hunting [25]. High human affluence on these roads could be causing the displacement of the species even in optimal habitat areas, as has been seen with other carnivore communities in response to activities such as hiking [52,53]. Interestingly, we found no relationship between roads and wildcat presence, even though other studies have demonstrated that traffic collisions are among the highest causes of mortality in this species [32,48,51].

In relation to elevation, some studies indicate that the optimal elevation for wildcats in Europe is between 300–800 m.a.s.l. and that this species avoids higher altitudes with greater snow persistence [54,55]. In contrast, our model indicates that higher elevation areas are more suitable for wildcats than low elevation areas, similar to the findings from southern Spain where wildcat detection was positively associated with elevation [35]. In Spain, snow persists only in the highest mountains, and over shorter periods, wildcats can stay inside the forest where the influence of the snow is lower and they can also move to lower areas looking for prey and refuge [55]. Climate change is leading to a rapid decrease in snow cover and persistence, which is already less frequent in southern countries such as Spain, so this phenomenon could be explaining the variations detected throughout the species’ range [56].

Most areas identified as potentially suitable habitats for wildcats were located in protected areas, which is not surprising given that protected areas in Asturias provide better forest cover than unprotected areas. Other studies show that wildcats are strongly associated with forests [48], likely due to the wildcats’ requirement of forest cover for shelter [57,58,59,60]. In addition, forest areas have a high number of rodents, the most important prey of wildcats, and thus provide good areas for hunting [32]. These results on the preference for forests stand on the other hand with other studies indicating a preference for mixed patches with farmland, shrublands, and grasslands [20,32]. Such apparent discrepancies may be explained by the fact that the habitat requirements of the wildcat are strongly conditioned by the availability of food [61], thus, this seems to indicate that the region’s forests have abundant prey. Interestingly, the resulting model also indicated a high probability of wildcat presence above the tree line (approximately 1700 m.a.s.l). Though such high elevation areas lack forest for cover, the scrubland habitat provides fertile hunting ground for rodents [62,63], which likely explains the high likelihood of wildcat presence and confirms the previous hypothesis.

This study identified suitable non-inhabited areas within the Asturias region, but possible expansion in the case of population growth could also extend outside the study area. The study population is part of a population continuum in the northern mountain range of the Iberian Peninsula connected to other high-density areas such as those of Central Spain or the Pyrenees, and thus, to the rest of Europe [32,64]. Much of the Iberian Peninsula is undergoing significant growth in forest covers due to reforestation and rural abandonment [65]. Growth in forest masses and the decrease in population density observed could lead to the expansion of the study species to new zones, in line with our results, and as has been seen in other vertebrate species [66,67]. Nevertheless, larger scale studies would be needed to accurately analyse the expansion potential of wildcats on a national scale if a strong population expansion occurs.

Identifying potential suitable habitats for wildcats and the strongest predictors of wildcat occurrence could allow authorities and conservation organisations to create effective management plans for this species. Expansion opportunities detected due to the availability of suitable areas could result in population sinks if the threats to the study species are not eliminated. In our model, only footpaths had a negative effect on wildcat distribution, so affluence on footpaths near breeding territories should be regulated, and the creation of new tracks should be avoided in order to maintain wildcat populations. Wildcat females have stricter habitat requirements than males, which demonstrates a higher tolerance to habitat fragmentation and human presence [68]. The integrity of wildcat populations is maintained when habitat conditions are favourable, even in contact with domestic cats [69,70]. So, if in the future, a conservation program for wildcats is needed, one of the priority actuations should be the conservation of their habitats. Future studies analysing drivers influencing wildcat distribution should also focus on monitoring their main preys, including their habitat requirements and how their abundance fluctuates over the year. Our findings should enhance the understanding and management of wildcat populations in Asturias, an important area for the species.

## 5. Conclusions

The results suggest that distribution in one of the main European wildcat populations is conditioned by the availability of habitats with abundant food and the potential negative effects associated with hunting or the high affluence of people on footpaths. The abundance of this species at high altitudes in the study area seems to be conditioned by low snowfall intensity in the south of its range, so climate change may be having an indirect positive effect on the distribution of the species. Targeted management to expand the current range of the species should focus on the regulation of activities that involve high affluence through critical areas, such as hunting or hiking.

## Figures and Tables

**Table 1:** Description, source, and original format of the 29 environmental variables used for the construction of Maxent models. Variables marked with * show no correlation (Pearson coefficient < 0.7) and were used in the models.

Name	Description	Source	Format	Year
Highways *	Length highways	BCN200	Vector	2015
Roads *	Length autonomic and national roads	BCN200	Vector	2015
Footpaths *	Length footpaths	BCN200	Vector	2015
Rivers *	Length rivers	BCN200	Vector	2015
Elevation and slope *		MDT200	Raster	2017
NDVI *	Normalised Difference Vegetation Index	Instituto de Recursos Naturales y Ordenación del Territorio (INDUROT)	Raster	2011
Land cover *	Percentage of each class per grid: cliffs, farmlands, fern, forests, gorse, heath, human structures, open grounds, pastures, forest plantations, shrublands, water, wetlands; n° classes, and Shannon index	Cartografía Temática Ambiental del Principado de Asturias 1989–1998 (1:50,000)	Vector	1998
Human density *		SADEI nomenclator	Vector	2015
Bio 1	Annual mean temperature	Worldclim	Raster	2000
Bio 5	Max temperature of warmest month	Worldclim	Raster	2000
Bio 6	Min temperature of coldest month	Worldclim	Raster	2000
Bio 7	Temperature annual range (BIO5–BIO6)	Worldclim	Raster	2000
Bio 12	Annual precipitation	Worldclim	Raster	2000
Bio 15 *	Precipitation seasonality (Coefficient of variation)	Worldclim	Raster	2000

**Table 2:** Evaluation metrics of the 5 candidate models with higher empirical support, built to evaluate the suitability for wildcat range in Asturias. (^a^) Feature types: L—linear, Q—quadratic, H—hinge, P—product, T—threshold.

Feature Types ^a^	Regularisation Multiplier	Full AUC	Mean AUC	AUC Diff	OR Min	AICc	Δ AICc	Nparam
LQT	3.5	0.840	0.750	0.093	0.128	1777.99	0	26
LQHT	1.5	0.870	0.792	0.094	0.085	1779.50	1.511	35
LQ	2	0.833	0.752	0.096	0.133	1781.71	3.718	25
LQ	4	0.830	0.746	0.092	0.128	1784.11	6.122	23
LQT	4.5	0.832	0.745	0.091	0.128	1784.53	6.548	24

**Table 3:** Variables of the MaxEnt model describing the probability of wildcat range. Percentage values are based on a heuristic method that estimates the proportional contribution of each variable to the model training gain for every iteration during model fitting.

Variable	Percentage Contribution
Elevation	39
Percent of forest	26.6
Footpaths	15.9
Percent of forest plantations	7.6
Slope	5.2
Precipitation seasonality	1.5
Percent of fern	1.4
Percent of shrubland	0.9
River density	0.8
Number of landcover classes	0.8
Percent of heath	0.2
Percent of pasture	0.1
Shannon index	0.1
Roads	0
Percent of human structures	0
Percent of gorse	0
Percent of open ground	0
NDVI	0
Human density	0
Highways	0
Percent of water	0
Percent of wetland	0
Percent of farmlands	0
Percent of cliffs	0

## Data Availability

Data used for this study contain sensitive information on a species potentially conflicting with human interests. Data may be provided upon request to the corresponding author when the reason is justified.

## Supplementary Materials

The following supporting information can be downloaded at: https://www.mdpi.com/article/10.3390/ani14182708/s1. Figure S1: Evaluation of the 130 candidate models of the distribution of the wildcat in Asturias. Evaluated based on regularisation multipliers from 0 to 10, and 5 types of parameterisations (linear (L), quadratic (Q), product (P), threshold (T), and hinge (H)) and their combinations. Figure S2: Result of the jackknife test of importance of the variables for the model. Figure S3: Associations between predicted suitability estimated from each of the included environmental predictors.

## References

[1] Davoli M., Ghoddousi A., Sabatini F.M., Fabbri E., Caniglia R., Kuemmerle T. (2022). Changing patterns of conflict between humans, carnivores and crop-raiding prey as large carnivores recolonize human-dominated landscapes. Biol. Conserv..

[2] Bombieri G., Penteriani V., Almasieh K., Ambarlı H., Ashrafzadeh M.R., Das C.S., Dharaiya N., Hoogesteijn R., Hoogesteijn A., Ikanda D. (2023). A worldwide perspective on large carnivore attacks on humans. PLOS Biol..

[3] Krofel M., Berce M., Berce T., Kryštufek B., Lamut S., Tarman J., Fležar U. (2023). New mesocarnivore at the doorstep of Central Europe: Historic development of golden jackal (*Canis aureus*) population in Slovenia. Mammal Res..

[4] Díaz-Ruiz F., Descalzo E., Martínez-Jauregui M., Soliño M., Márquez A.L., Farfán M.Á., Real R., Ferreras P., Delibes-Mateos M. (2024). Combining ranger records and biogeographical models to identify the current and potential distribution of an expanding mesocarnivore in southern Europe. Sci. Total Environ..

[5] Benson J.F., Mahoney P.J., Sikich J.A., Serieys L.E.K., Pollinger J.P., Ernest H.B., Riley S.P.D. (2016). Interactions between demography, genetics, and landscape connectivity increase extinction probability for a small population of large carnivores in a major metropolitan area. Proc. R. Soc. B Biol. Sci..

[6] Boudreau M.R., Gantchoff M.G., Ramirez-Reyes C., Conlee L., Belant J.L., Iglay R.B. (2022). Using habitat suitability and landscape connectivity in the spatial prioritization of public outreach and management during carnivore recolonization. J. Appl. Ecol..

[7] Planillo A., Wenzler-Meya M., Reinhardt I., Kluth G., Michler F.U., Stier N., Louvrier J., Steyer K., Gillich B., Rieger S. (2024). Understanding habitat selection of range-expanding populations of large carnivores: 20 years of grey wolves (*Canis lupus*) recolonizing Germany. Divers. Distrib..

[8] Phillips S.J., Anderson R.P., Dudík M., Schapire R.E., Blair M.E. (2017). Opening the black box: An open-source release of Maxent. Ecography.

[9] Zarzo-Arias A., Penteriani V., del Mar Delgado M., Torre P.P., García-González R., Mateo-Sánchez M.C., García P.V., Dalerum F. (2019). Identifying potential areas of expansion for the endangered brown bear (*Ursus arctos*) population in the Cantabrian Mountains (NW Spain). PLoS ONE.

[10] Su H., Bista M., Li M. (2021). Mapping habitat suitability for Asiatic black bear and red panda in Makalu Barun National Park of Nepal from Maxent and GARP models. Sci. Rep..

[11] Schmidt K., Górny M., Jędrzejewski W. (2023). Effect of microhabitat characteristics for predicting habitat suitability for a stalking large carnivore—The Eurasian lynx in middle Europe. Anim. Conserv..

[12] Nowell K., Jackson P. (1996). Wild Cats: Status Survey and Conservation Action Plan.

[13] Macdonald D.W. (2004). The Scottish Wildcat: Analyses for Conservation and an Action Plan.

[14] Bastianelli M.L., Premier J., Herrmann M., Anile S., Monterroso P., Kuemmerle T., Dormann C.F., Streif S., Jerosch S., Götz M. (2021). Survival and cause-specific mortality of European wildcat (*Felis silvestris*) across Europe. Biol. Conserv..

[15] Gil-Sánchez J.M., Barea-Azcón J.M., Jaramillo J., Herrera-Sánchez F.J., Jiménez J., Virgós E. (2020). Fragmentation and low density as major conservation challenges for the southernmost populations of the European wildcat. PLoS ONE.

[16] Slotta-Bachmayr L., Meikl M., Hagenstein I. (2016). Current status of the European wildcat (*Felis silvestris silvestris*, Schreber, 1777) in Austria. Acta ZooBot Austria.

[17] Gerngross P., Ambarli H., Angelici F.M., Anile S., Campbell R., Ferreras de Andres P., Gil-Sanchez J.M., Götz M., Jerosch S., Mengüllüoglu D. (2023). Felis silvestris (Amended Version of 2022 Assessment). The IUCN Red List of Threatened Species 2023: e.T181049859A224982454. https://www.worldclim.org.

[18] Steyer K., Tiesmeyer A., Muñoz-Fuentes V., Nowak C. (2018). Low rates of hybridization between European wildcats and domestic cats in a human-dominated landscape. Ecol. Evol..

[19] Migli D., Astaras C., Boutsis G., Diakou A., Karantanis N.E., Youlatos D. (2021). Spatial ecology and diel activity of European Wildcat (*Felis silvestris*) in a protected lowland area in Northern Greece. Animals.

[20] Ruiz-Villar H., Bastianelli M.L., Heurich M., Anile S., Díaz-Ruiz F., Ferreras P., Götz M., Herrmann M., Jerosch S., Jubete F. (2023). Agriculture intensity and landscape configuration influence the spatial use of wildcats across Europe. Biol. Conserv..

[21] Monterroso P., Célio Alves P., Ferreras P. (2014). Plasticity in circadian activity patterns of mesocarnivores in Southwestern Europe: Implications for species coexistence. Behav. Ecol. Sociobiol..

[22] Martín-Díaz P., Gil-Sánchez J.M., Balleseros-Duperón E., Barea-Azcón J.M., Virgós E., Pardavila X., Moleón M. (2018). Integrating space and time in predator-prey studies: The case of wildcats and rabbits in SE Spain. Mamm. Biol..

[23] Jiménez-Albarral J.J., Urra F., Jubete F., Roman J., Revilla E., Palomares F. (2021). Abundance and use pattern of wildcats of ancient human-modified cattle pastures in northern Iberian Peninsula. Eur. J. Wildl. Res..

[24] Ruiz-Villar H., Jubete F., Revilla E., Román J., Urra F., López-Bao J.V., Palomares F. (2021). Like cat and fox: Diurnal interactions between two sympatric carnivores in pastoral landscapes of NW Spain. Eur. J. Wildl. Res..

[25] Ruiz-Villar H., Morales-González A., López-Bao J.V., Palomares F. (2024). Humans and traffic influence European wildcat behaviour in pastoral landscapes. Anim. Behav..

[26] Széles G.L., Purger J.J., Molnár T., Lanszki J. (2018). Comparative analysis of the diet of feral and house cats and wildcat in Europe. Mammal Res..

[27] Soto C.A., Palomares F. (2014). Surprising low abundance of European wildcats in a mediterranean protected area of Southwestern Spain. Mammalia.

[28] Lozano J., Virgós E., Cabezas-Díaz S. (2013). Monitoring European wildcat *Felis silvestris* populations using scat surveys in central Spain: Are population trends related to wild rabbit dynamics or to landscape features?. Zool. Stud..

[29] Quevedo M., Echegaray J., Fernández-Gil A., Leonard J.A., Naves J., Ordiz A., Revilla E., Vilà C. (2019). Lethal management may hinder population recovery in Iberian wolves. Biodivers. Conserv..

[30] Martínez Cano I., Taboada F.G., Naves J., Fernández-Gil A., Wiegand T. (2016). Decline and recovery of a large carnivore: Environmental change and long-term trends in an endangered brown bear population. Proc. R. Soc. B Biol. Sci..

[31] Kramer-Schadt S., Niedballa J., Pilgrim J.D., Schröder B., Lindenborn J., Reinfelder V., Stillfried M., Heckmann I., Scharf A.K., Augeri D.M. (2013). The importance of correcting for sampling bias in MaxEnt species distribution models. Divers. Distrib..

[32] Lozano J., Salvador A. (2017). Gato montés—*Felis silvestris* Schreber, 1777. Enciclopedia Virtual de los Vertebrados Españoles.

[33] Eriksson T., Dalerum F. (2018). Identifying potential areas for an expanding wolf population in Sweden. Biol. Conserv..

[34] Lozano J. (2010). Habitat use by European wildcats (*Felis silvestris*) in central Spain: What is the relative importance of forest variables?. Anim. Biodivers. Conserv..

[35] Carreras-Duro J., Moleón M., Barea-Azcón J.M., Ballesteros-Duperón E., Virgós E. (2016). Optimization of sampling effort in carnivore surveys based on signs: A regional-scale study in a Mediterranean area. Mamm. Biol.-Z. Saugetierkd..

[36] Cushman S.A., Kilshaw K., Kaszta Z., Campbell R.D., Gaywood M., Macdonald D.W. (2024). Variable importance and scale of influence across individual scottish wildcat hybrid habitat models. Ecol. Model..

[37] Garcia-Perea R. (2007). Felis silvestris Schreber, 1777. Atlas y Libro Rojo de los Mamıferos Terrestres de España.

[38] Phillips S.J., Dudík M., Schapire R.E., Maxent Software for Modeling Species Niches and Distributions (2017). In (Version 3.4.0). http://biodiversityinformatics.amnh.org/open_source/maxent.

[39] Hijmans R.J., Phillips S., Leathwick J.R., Elith J. (2017). Package ‘dismo’. Circles.

[40] Muscarella R., Galante P.J., Soley-Guardia M., Boria R.A., Kass J.M., Uriarte M., Anderson R.P. (2014). ENMeval: An R package for conducting spatially independent evaluations and estimating optimal model complexity for Maxent ecological niche models. Methods Ecol. Evol..

[41] Warren D.L., Seifert S.N. (2011). Ecological niche modeling in Maxent: The importance of model complexity and the performance of model selection criteria. Ecol. Appl..

[42] Akaike H. (1974). A new look at the statistical model identification. IEEE Trans. Autom. Control.

[43] Burnham K.P., Anderson D.R. (2002). Model Selection and Multimodel Inference: A Practical Information-Theoretic Approach.

[44] Fielding A.H., Bell J.F. (1997). A review of methods for the assessment of prediction errors in conservation presence/absence models. Environ. Conserv..

[45] Araújo M.B., Pearson R.G., Thuiller W., Erhard M. (2005). Validation of species–climate impact models under climate change. Glob. Chang. Biol..

[46] Phillips S.J., Anderson R.T., Schapire R.E. (2006). Maximum entropy modeling of species geographic distributions. Ecol. Model..

[47] Young N., Carter L., Evangelista P. (2011). A MaxEnt Model v3. 3.3 e Tutorial (ArcGIS v10).

[48] Klar N., Fernández N., Kramer-Schadt S., Herrmann M., Trinzen M., Büttner I., Niemitz C. (2008). Habitat selection models for European wildcat conservation. Biol. Conserv..

[49] Virgós E., Travaini A. (2005). Relationship between small-game hunting and carnivore diversity in Central Spain. Biodivers. Conserv..

[50] Cano C., Ayerza P., de la Hoz J.F. (2006). El Veneno en España (1990–2005). Análisis del Problema, Incidencia y Causas. Propuestas de WWF/Adena.

[51] Urra F. (2003). El Gato Montés en Navarra: Distribución, Ecología y Conservación.

[52] Baker A.D., Leberg P.L. (2018). Impacts of human recreation on carnivores in protected areas. PLoS ONE.

[53] Bandak S., Sarno R., Peterson M., Farkas D., Grigione M. (2020). Active humans, inactive carnivores, and hiking trails within a suburban preserve. Suburb. Sustain..

[54] Liberek M. (1999). Eco-ethologie du chat sauvage *Felis s. silvestris*, Schreber 1777 dans le Jura Vaudois (Suisse): Influence de la Couverture Neigeuse. Ph.D. Thesis.

[55] Mermod C.P., Liberek M. (2002). The role of snowcover for European wildcat in Switzerland. Z. Jagdwiss..

[56] Lastrada E., Garzón-Roca J., Cobos G., Torrijo F.J. (2021). A decrease in the regulatory effect of snow-related phenomena in spanish mountain areas due to climate change. Water.

[57] Parent G.H. (1975). La migration récente, à caractère invasionnel, du chat sauvage, *Felis silvestris silvestris* Schreber, en Lorraine Belge. Mammalia.

[58] Piechocki R. (1990). Die Wildkatze: Felis silvestris Wolf, Verlagskg.

[59] Stahl P. (1986). Le Chat Forestier d’Europe (*Felis silvestris*, Schreber 1777): Exploitation des Ressources et Organisation Spatiale. Ph.D. Thesis.

[60] Wittmer H.U. (2001). Home range size, movements, and habitat utilization of three male European wildcats (*Felis silvestris* Schreber, 1777) in Saarland and Rheinland-Pfalz (Germany). Mamm. Biol..

[61] Silva A.P., Kilshaw K., Johnson P.J., MacDonald D.W., Rosalino L.M. (2013). Wildcat occurrence in Scotland: Food really matters. Divers. Distrib..

[62] Osbourne J.D., Anderson J.T., Spurgeon A.B. (2005). Effects of habitat on small-mammal diversity and abundance in West Virginia. Wildl. Soc. Bull..

[63] Sullivan T.P., Sullivan D.S. (2006). Plant and small mammal diversity in orchard versus non-crop habitats. Agric. Ecosyst. Environ..

[64] López-Martín J.M., García F.J., Duch A., Virgós E., Lozano J., Duarte J., España A.J. (2007). Felis silvestris Schreber, 1777. Atlas y Libro Rojo de los Mamíferos de España.

[65] Vila Subirós J., Rodríguez-Carreras R., Varga D., Ribas A., Úbeda X., Asperó F., Llausàs A., Outeiro L. (2016). Stakeholder perceptions of landscape changes in the Mediterranean mountains of the North-Eastern Iberian Peninsula. Land Degrad. Dev..

[66] Regos A., Domínguez J., Gil-Tena A., Brotons L., Ninyerola M., Pons X. (2016). Rural abandoned landscapes and bird assemblages: Winners and losers in the rewilding of a marginal mountain area (NW Spain). Reg. Environ. Chang..

[67] Martínez-Abraín A., Jiménez J., Jiménez I., Ferrer X., Llaneza L., Ferrer M., Palomera G., Ballesteros F., Galán P., Oro D. (2020). Ecological consequences of human depopulation of rural areas on wildlife: A unifying perspective. Biol. Conserv..

[68] Oliveira T., Urra F., López-Martín J.M., Ballesteros-Duperón E., Barea-Azcón J.M., Moléon M., Gil-Sánchez J.M., Alves P.C., Díaz-Ruíz F., Ferreras P. (2018). Females know better: Sex-biased habitat selection by the European wildcat. Ecol. Evol..

[69] Oliveira R., Godinho R., Randi E., Alves P.C. (2008). Hybridization versus conservation: Are domestic cats threatening the genetic integrity of wildcats (*Felis silvestris silvestris*) in Iberian Peninsula?. Philos. Trans. R. Soc. B Biol. Sci..

[70] Gil-Sánchez J.M., Jaramillo J., Barea-Azcón J.M. (2015). Strong spatial segregation between wildcats and domestic cats may explain low hybridization rates on the Iberian Peninsula. Zoology.

