# Supplementary material for: Modelling the Distribution and Habitat Suitability of the European Wildcat (Felis silvestris) in North-Western Spain and Its Conservation Implications
Source: Animals (Basel). 2024 Sep 18;14(18):2708. doi: 10.3390/ani14182708 (PMC11429425; doi:10.3390/ani14182708)
Supplement: Supplementary file 1 [file animals-14-02708-s001.zip › animals-3160886-supplementary.pdf]

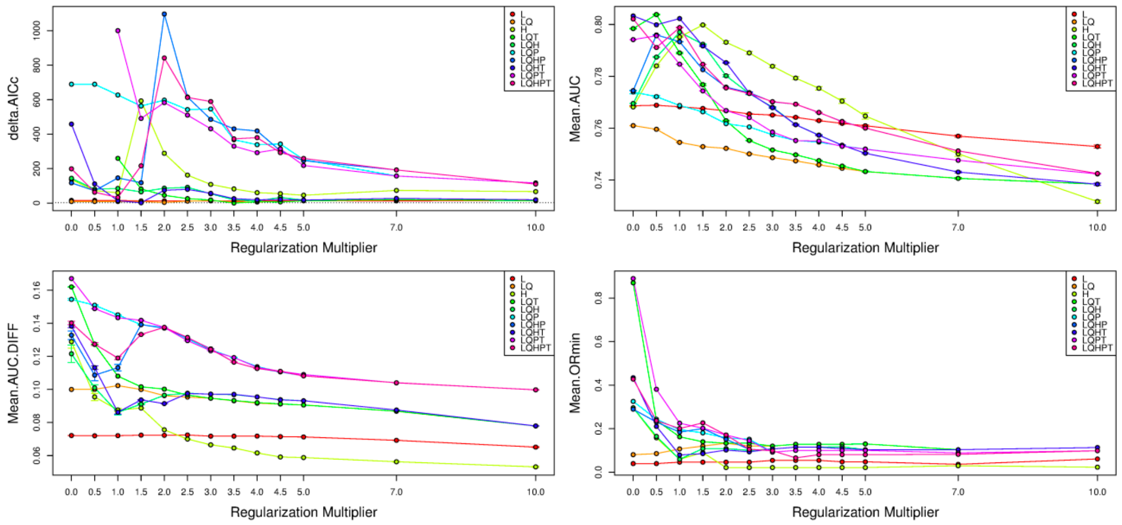

Figure S1. Evaluation of the 130 candidate models of the distribution of the wildcat in Asturias. Evaluated based on regularization multipliers from 0 to 10 and 5 types of parameterizations (linear (L), quadratic (Q), product (P), threshold (T) and hinge (H)) and their combinations.

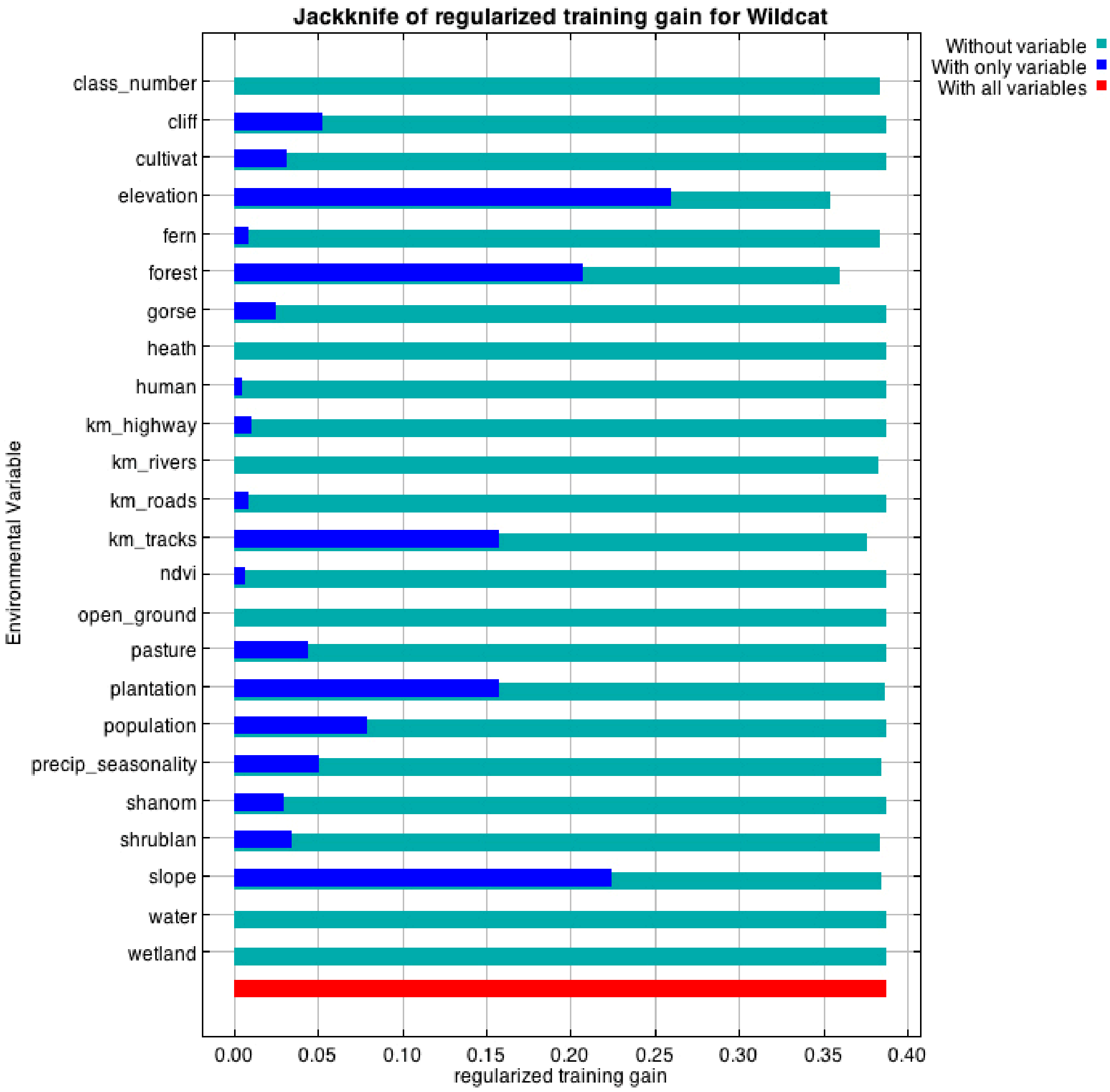

Figure S2. Result of the jackknife test of importance of the variables for the model.

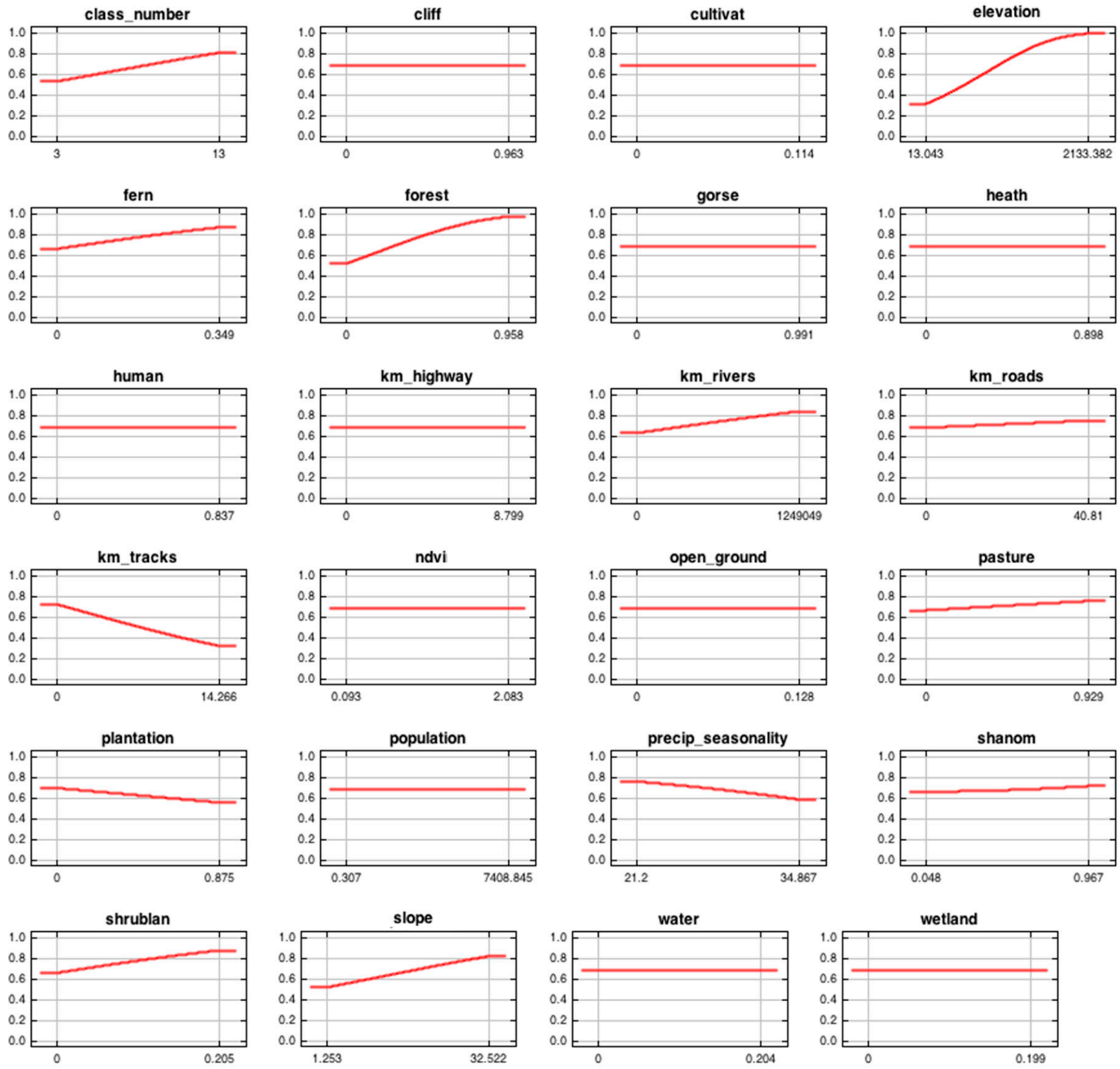

Figure S3. Associations between predicted suitability estimated from each of the included environmental predictors.
